# Supplementary material for: Is the number of previous hospitalizations associated with increased in-hospital mortality after hip fracture in a developing country?
Source: PLoS One. 2020 Oct 9;15(10):e0240229. doi: 10.1371/journal.pone.0240229 (PMC7546455; doi:10.1371/journal.pone.0240229)
Supplement: Attachment — Submitted filename: Paper_14_08_2019.docx [file pone.0240229.s002.docx]

**Is the number of previous hospitalizations associated with increased in-hospital mortality after hip fracture in a developing country?**

Helen Regina Mota Machareth de Morais^1*^, Edison Iglesias de Oliveira Vidal^2^, Claudia Medina Coei^1^, Rejane Sobrino Pinheiro^1^.

^1^ Institute for Collective Health Studies, Federal University of Rio de Janeiro, Rio de Janeiro, Rio de Janeiro, Brazil.

^2^ Botucatu Medical School, Sao Paulo State University (UNESP), Botucatu, São Paulo, Brazil.

*Corresponding Author:

E-mail: helenmachareth@gmail.com (HRMMM)

**Abstract**

We aimed to examine whether the number of previous hospitalizations and the main diagnoses of those hospitalizations are associated with increased in-hospital mortality. That assessment is relevant because if those variables are shown to be associated with increased mortality, that finding could support their use as proxies for comorbidity burden for case mix adjustment in statistical models seeking to compare the performance of hospitals regarding hip fracture mortality in settings with limited hospital information systems. Retrospective cohort study of all public hospital admissions for older adults with hip fractures in the city of Rio de Janeiro between 2010 and 2011. We used logistic regression methods to examine the association between in-hospital mortality and the number of hospitalizations in the previous two years and their main diagnoses. Among 1938 patients included in the study there were 103 (5.3%) in-hospital deaths. Although the presence of previous hospitalizations in the previous two years was associated with increased mortality (OR: 1.72, 95%CI: 1.03 to 2.86) we did not find evidence of a gradient of increased mortality with growing number of previous hospitalizations. Additionally, several diseases recorded as main diagnoses of previous hospitalizations were not associated with increased mortality rates as was expected. Our results suggest that, in settings where local hospital information systems have limited access to secondary diagnoses, the use of the number of previous hospitalizations or the main diagnoses associated with those hospitalizations as proxies for the profile of comorbidities of older adults with hip fractures may not be an effective way to adjust for case mix when comparing in-hospital mortality rates among hospitals.

**Introduction**

The quality of health services must be constantly evaluated and monitored in order to optimize their impact on population health. Health service quality encompasses aspects such as access to services, equity, technical adequacy, effectiveness, costs, safety and patient satisfaction [1]. Performance indicators are useful when evaluating health service quality because they gauge the care process as well as its favorable or unfavorable impact on individuals’ health [1]. Performance indicators are instruments that seek to monitor the quality of health care services and work processes so as to identify the need for more specific evaluation, when potential problems are identified [2].

In-hospital mortality after a hip fracture is an important indicator of quality of care for a common condition that affects older adults around the world. It is considered a performance indicator and may be used in evaluating a single hospital’s quality over time or in comparing several hospitals [2-4]. Indeed, in-hospital mortality after a hip fracture is among the 26 quality indicators that the Agency for Healthcare Research and Quality determined for the assessment of care in American hospitals [5].

Reviews of predictive factors for death following a hip fracture point to a strong relationship between death and factors such as male sex, advanced age, number of comorbidities and the presence of cognitive deficit [6-9]. Comorbidities most closely associated with death in these studies were pulmonary diseases, heart diseases, kidney diseases, diabetes and stroke [6-8]. Previous hospitalizations have also been considered to be associated with increased mortality following hip fracture [10-11].

For in-hospital mortality after hip fracture in older adults to be used as a quality indicator to compare the performance of hospitals there are important factors that must be taken into account so that differences in findings do not primarily reflect differences in case mix among hospitals, but rather the quality of care provided in those institutions [11-14]. Risk adjustment aims to reduce the confounding role that some variables related to case mix, such as patients’ burden of comorbidities, functional and socioeconomic status, may play on health outcomes that are used as markers of quality of care among institutions [14-18].

In Brazil, hospitalization data from the Brazilian National Health System are recorded in an administrative database, the Hospital Admission Information System, which is the most important national source of information for planning and monitoring hospital care in the country. Additionally, the national Hospital Admission Information System may serve as an instrument for the assessment of the quality of inpatient care. However, up to this moment the Brazilian Hospital Admission Information System still allows the recording of only one comorbidity and this information has been historically recorded poorly [14]. This is an important limitation of the Brazilian Hospital Admission Information System, which is shared by information systems from several other developing countries [19]. A possible strategy to attempt to overcome that important shortcoming for analyses related to the quality of care is to use data from previous hospitalizations routinely recorded in Hospital Admission Information Systems, such as the number of previous hospitalizations and the main diagnosis for each hospitalization episode, as a tool for risk adjustment in statistical models.

This study aims to analyze the association between previous hospital admissions and in-hospital mortality in older adults who underwent surgical repair of a hip fracture. It also seeks to consider the association of main diagnoses of previous hospitalizations and in-hospital death following a hip fracture. The presence of significant associations between previous hospitalizations and/or their main diagnoses and in-hospital mortality after a hip fracture could represent evidence favoring the use of those variables for risk adjustment using administrative data in scenarios typical of several developing countries, where hospital databases suffer from major shortcomings regarding the registry of comorbidities and secondary diagnoses.

**Methods**

This is a non-concurrent cohort study of a population of elderly patients hospitalized for hip fractures in the city of Rio de Janeiro, Brazil, between 2010 and 2011. We analyzed data from the national Hospital Admission Information System for patients aged 62 years and older, whose main diagnostic codes for hospital admission according to 10^th^ revision of the International Classification of Diseases (ICD10) were fracture of the neck of femur (S72.0), pertrochantheric fracture (S72.1) and subtrochanteric fracture (S72.2) and who underwent surgical treatment of fracture.

Causes of previous hospitalization were identified through the main diagnosis recorded in the national Hospital Admission Information System for hospitalizations that took place up to two years prior to the hospitalization for the index hip fracture. We recovered this information through probabilistic record linkage of the Hospital Admission Information System data from patients aged 62 years and older hospitalized for hip fractures in 2010 and 2011 and the same database for patients aged 60 years and older hospitalized between 2008 and 2011 for any cause.

We used a five-stage probabilistic record linkage technique, following a strategy recommended by Camargo Jr. and Coeli [20-21] and using the OpenReclink software (version: 3.1) (http://reclink.sourceforge.net/). Previous research in a similar setting showed 99.4% specificity, 85.5% sensibility, 98.1% positive predictive value and 94.9% negative predictive value for correct matching of records using this methodological approach [22].

Access to those databases granted by the Rio de Janeiro Municipal Health Secretary, after approval by the Ethics Review Boards from the Institute for Collective Health Studies and the Municipal Health Secretary, under processes 1.052.216 and 600.963-0, respectively. (please attach this approval)

We analyzed the association between in-hospital mortality after a hip fracture and previous hospitalizations based on three ways of classifying the latter: existence of previous hospitalizations (yes or no); number of previous hospitalizations; and main diagnosis of previous hospitalizations. For the last classification, we grouped main diagnoses based on the scientific literature on factors that impact mortality following hip fracture [6-8, 18].

We used Pearson’s chi-square test to evaluate the association between the dependent variable (in-hospital mortality) and the following variables in univariate analyses: presence and number of previous hospitalizations within the last two years, sex, age (classified in age groups from 60 to 69 years, 70 to 79 years, 80 to 89 years and 90 years or older) and type of hip fracture (classified as femoral neck fracture, pertrochantheric fracture and subtrochanteric fracture). We calculated the odds ratio of in-hospital death associated with previous hospitalizations through two multiple logistic regression models [18], adjusted for sex, age and type of fracture. In one model the occurrence of previous hospitalizations over the last two years was coded as a dichotomous variable and in the other model as the total number of previous hospitalizations within that same time frame. We did not adjust those regression models for the number or type of main diagnoses from previous hospitalizations because too much collinearity would be expected with the variable encoding the number of previous hospitalizations.

We also assessed the association between in-hospital mortality after a hip fracture and the different causes of previous hospitalizations by means of simple and multiple logistical regressions. For the multiple regression model all groups of causes of previous hospitalizations were included and adjusted for patients’ sex, age and type of fracture. We used the Stata9® software to perform all analyses.

**Ethical approval**

The Ethical Review Committees of the Institute for Collective Health Studies, and the Municipal Health Secretary, approved this study, under the processes 1.052.216 and 600.963-0.

**Results**

There were 2046 records of patients aged 62 years and older admitted to hospitals of the Brazilian National Health System because of a hip fracture between 2010 and 2011 in the city of Rio de Janeiro. We excluded 94 (4.59%) patients because they had not been submitted to surgical repair of the fracture and 14 (0.68%) patients because their hip fracture resulted from multiple high-intensity trauma, such as that from a car accident. The mean age of the 1938 patients included was 79.4 years (SD: 8.3). The median length of stay of patients was 15 days (interquartile range: 10 – 22 days). In total, there were 103 episodes of in-hospital death corresponding to 5.3% mortality rate. Table 1 displays information regarding the distribution of patients regarding sex, age groups, type of fracture and number of hospitalizations both in overall terms and concerning the occurrence of in-hospital death. Table 2 shows the frequency of main diagnoses recorded for previous hospitalizations episodes. Table 3 displays the results of the two multiple logistic regression models examining the association between in-hospital mortality and previous hospitalizations within the last two years. We also performed a sensitivity analysis by reframing the classification of the number of previous hospitalizations into three categories: 0, 1 and 2 or more episodes of hospitalizations. The overall results of that sensitivity analysis were not significantly different from those presented in table 3. Table 4 shows the results of simple and multiple logistic regression models examining the association between main diagnoses from previous hospitalizations within the last two years and in-hospital death after a hip fracture.

| **Table 1.** Comparison of patient characteristics according to the occurrence of in-hospital mortality after a hip fracture. | | | | | |
| --- | --- | --- | --- | --- | --- |
|  |  |  |  |  |  |
| **Variable** | **Total** | | **Death** | | ***P value**** |
|  | **n** | **%** | **n** | **%** |  |
| **Sex** |  |  |  |  | 0.65 |
| Female | 1411 | 72.8 | 77 | 5.5 |  |
| Male | 527 | 27.2 | 26 | 4.9 |  |
| **Age (years)** |  |  |  |  | < 0.01 |
| 60 – 69 | 270 | 13.9 | 5 | 1.9 |  |
| 70 – 79 | 658 | 33.9 | 20 | 3.0 |  |
| 80 – 89 | 788 | 40.7 | 46 | 5.8 |  |
| ≥ 90 | 222 | 11.5 | 32 | 14.4 |  |
| **Fracture Type** |  |  |  |  | 0.04 |
| Cervical | 1098 | 56.7 | 71 | 6.5 |  |
| Pertrochantheric | 619 | 31.9 | 23 | 3.7 |  |
| Subtrochanteric | 221 | 11.4 | 9 | 4.1 |  |
| **Previous Hospitalization** |  |  |  |  | 0.11 |
| No | 1648 | 85.0 | 82 | 5.0 |  |
| Yes | 290 | 15.0 | 21 | 7.2 |  |
| **Number of Previous Hospitalization** |  |  |  |  | 0.75 |
| 0 | 1648 | 85.0 | 82 | 5.0 |  |
| 1 | 214 | 11.0 | 16 | 7.5 |  |
| 2 | 53 | 2.7 | 4 | 7.5 |  |
| 3 | 9 | 0.5 | 0 | 0.0 |  |
| 4 | 9 | 0.5 | 1 | 11.1 |  |
| ≥ 5 | 5 | 0.3 | 0 | 0.0 |  |
| **Total** | 1938 | 100.0 | 103 | 5.3 |  |
| * χ² test |  |  |  |  |  |

| **Table 2**. Main diagnoses recorded for hospitalization episodes within the two years preceding the index hospitalization due to a hip fracture. | | | |
| --- | --- | --- | --- |
| **Diagnoses** | **n** | **%** |  |
| **Fractures** |  |  |  |
| Hip fracture | 20 | 6.9 |  |
| Other fractures and lesions | 78 | 26.9 |  |
| **Cardiovascular** |  |  |  |
| Cerebrovascular disease | 11 | 3.8 |  |
| Hypertensive heart disease | 7 | 2.4 |  |
| Arrhythmias and conduction disorders | 6 | 2.1 |  |
| Ischemic heart diseases | 4 | 1.4 |  |
| Peripheral Vascular Disease | 8 | 2.8 |  |
| Cardiac valve disease and congestive heart failure | 7 | 2.4 |  |
| **Neoplasms** |  |  |  |
| Lymphoma and leukemia | 9 | 3.1 |  |
| Malignant neoplasms | 18 | 6.2 |  |
| **Urinary System** |  |  |  |
| Severe and moderate kidney disease | 4 | 1.4 |  |
| Urinary tract infections | 18 | 6.2 |  |
| **Respiratory System** |  |  |  |
| Pneumonia | 12 | 4.1 |  |
| Tuberculosis | 3 | 1.0 |  |
| Chronic obstructive pulmonary disease | 4 | 1.4 |  |
| **Metabolic, Nutritional and Hematologic Diseases** |  |  |  |
| Anemias | 6 | 2.1 |  |
| Diabetes mellitus | 4 | 1.4 |  |
| Other metabolic, nutritional and hematologic diseases | 7 | 2.4 |  |
| **Other** |  |  |  |
| Chronic liver disease | 1 | 0.3 |  |
| Connective tissue disease | 6 | 2.1 |  |
| Infectious, gastrointestinal and other diseases | 41 | 14.1 |  |
| Schizophrenia | 1 | 0.3 |  |
| Vision disorders | 13 | 4.5 |  |
| Ulcers | 2 | 0.7 |  |
| **Total** | **290** | **100** |  |

| **Table 3.** Simple and multiple logistic regression models assessing in-hospital mortality after a hip fracture. Multiple logistic regression models were adjusted for sex, age and type of fracture. | | |
| --- | --- | --- |
| **Variables** | **Crude OR (95% CI)** | **Adjusted OR (95% CI)** |
| **Sex** |  |  |
| Male | 0.90 (0.57 to 1.42) | 1.08 (0.68 to 1.73) |
| Female | 1.00 | 1.00 |
| **Age (years)** |  |  |
| 60 - 69 | 1.00 | 1.00 |
| 70 - 79 | 1.66 (0.62 to 4.47) | 1.72 (0.63 to 4.64) |
| 80 - 89 | 3.29 (1.29 to 8.36) | 3.64 (1.42 to 9.31) |
| ≥ 90 | 8.93 (3.42 to 23.33) | 10.27 (3.89 to 27.13) |
| **Fracture Type** |  |  |
| Femoral neck | 1.00 | 1.00 |
| Pertrochanteric | 0.56 (0.35 to 0.90) | 0.46 (0.28 to 0.75) |
| Subtrochanteric | 0.61(0.30 to 1.25) | 0.58 (0.28 to 1.18) |
| **Previous Hospitalizations*** |  |  |
| Yes | 1.49 (0.91 to 2.45) | 1.72 (1.03 to 2.86) |
| No | 1.00 | 1.00 |
| **Number of Previous Hospitalizations*** |  |  |
| 0 | 1.00 | 1.00 |
| 1 | 1.54 (0.89 to 2.69) | 1.70 (0.95 to 2.99) |
| 2 | 1.56 (0.55 to 4.42) | 2.16 (0.74 to 6.29) |
| ≥ 3 | 0.87 (0.12 to 6.52) | 1.10 (0.14 to 8.66) |
| OR: odds ratio; CI: confidence interval. | | |
| * The OR of previous hospitalization was adjusted for sex, age and type of fracture, as well as the OR of number of previous hospitalizations. | | |

| **Table 4.** Simple and multiple logistic regression models for in-hospital mortality after a hip fracture, according to causes of previous hospitalization within the last two years. Multiple logistic regression models were adjusted for sex, age and type of fracture. | | |
| --- | --- | --- |
| **Variable** | **Crude OR**  **(95% CI)** | **Adjusted OR**  **(95% CI)** |
| Cerebrovascular diseases | 1.79  (0.23 to 14.11) | 3.10  (0.37 to 25.92) |
| Severe and moderate Kidney disease | 18.15  (2.53 to 130.15) | 25.45  (3.33 to 194.77) |
| Cardiac valve disease and congestive heart failure | 2.99  (0.36 to 25.06) | 2.83  (0.30 to 27.08) |
| Urinary tract infections | 2.25  (0.51 to 9.92) | 2.65  (0.58 to 11.98) |
| Infectious and gastrointestinal diseases | 0.44  (0.60 to 3.23) | 0.64  (0.06 to 4.74) |
| Ischemic heart diseases | 5.99  (0.62 to 58.06) | 10.55  (1.03 to 108.31) |
| Metabolic, nutritional and hematologic diseases | 7.25  (1.39 to 37.81) | 7.11  (1.26 to 39.94) |
| Other fractures and lesions | 1.81  (0.81 to 4.04) | 1.98  (0.87 to 4.51) |
| Neoplasms | 1.05  (0.14 to 7.96) | 1.31  (0.17 to 10.14) |
| Pneumonia | 3.61  (0.78 to 16.71) | 4.25  (0.83 to 21.80) |
| OR: odds ratio; CI: confidence interval. |  |  |
| *Due to a lack of variability, we were not able to estimate the OR of the following causes: anemias, arrhythmias and conduction disorders, diabetes mellitus, chronic liver disease, chronic obstructive pulmonary disease, peripheral vascular disease, connective tissue disease, schizophrenia, hip fracture, lymphoma and leukemia, tuberculosis, ulcers, prostatic hyperplasia and vision disorders. | | |

**Discussion**

This is one of the largest epidemiological studies examining in-hospital hip fracture mortality in Brazil [23-28]. Our study aimed to analyze the association between previous hospitalizations and their main diagnoses with in-hospital mortality after hip fracture surgery in older adults. Our main finding was that the occurrence of previous hospitalizations within the last two years was significantly associated with in-hospital mortality but paradoxically the number of hospitalizations in that period was not associated with a gradient of increased in-hospital mortality. On the one hand, the low number of individuals who had been hospitalized three or more times in our cohort could provide an explanation for this phenomenon because of lack of statistical power. On the other hand, the low number of individuals in that category might also be pointing towards the presence of selection bias, where individuals with worse health status are denied surgical treatment or even hospitalization for hip fracture, as has been discussed by others [29-30].The latter hypothesis is especially plausible given the fact that about 5% of the original sample were excluded because patients did not receive surgical treatment. Hence, our results suggest that the number of hospitalizations may not be a good proxy of the health status of older patients undergoing hip fracture surgery for risk adjustment in statistical models in contexts prone to the possibility of selection bias. Therefore, previous hospitalizations should be used with caution for risk adjustment of in-hospital mortality after hip fracture surgery in settings of Hospital Admission Information Systems with limited availability of data regarding comorbidities and secondary diagnoses.

To some extent, our findings disagree with those of other studies [10-11]. For instance, Meyer et al. from Oslo, Norway, [10] found that among 248 older adults hospitalized due to a hip fracture patients who had two or more hospitalizations in the two years preceding the hip fracture were four times more likely to die within one year than patients who had not been previously hospitalized. However, that study identified a clear dose-response gradient with higher odds of mortality for those with two or more hospitalizations than for that one episode of hospital admission. Aigner et al. [11] from Germany also observed that the odds of dying within six months and one year after a hip fracture was approximately two times higher in patients who had been hospitalized within the three months preceding the index hip fracture when compared with patients who had not had a similar experience. However, that study did not assess for the presence of dose-response gradient regarding mortality and their analyses did not show statistically significant differences regarding in-hospital mortality between those who had been hospitalized within three months before fracture and those who did not.

Regarding our assessment of causes of previous hospitalizations as a proxy for patients’ comorbidities, our results disclosed significant associations with in-hospital mortality only for three disease groups, namely severe and moderate kidney disease, ischemic heart diseases, and metabolic, nutritional and hematologic diseases. Although those associations are in agreement with the medical literature concerning risk factors for mortality after a hip fracture, the finding of no association between other equally relevant disease groups and mortality and the very absence of other known risk factors for mortality (e.g. dementia) are not [6-8, 18, 31-33] and point towards the inherent limitations of using main diagnoses of previous hospitalizations as a proxy and single source of information about the burden of comorbidities of patients. Such phenomenon is likely to have occurred because the process of ascertaining a main diagnosis for an episode of hospitalization usually favors acute diagnoses over more chronic baseline comorbidities. For instance, an older adult suffering from dementia who was hospitalized because of aspiration pneumonia will usually have the latter diagnosis recorded as the main diagnosis for hospital admission instead of dementia.

This study has several limitations. Our analyses were restricted to hospitalizations funded by the Brazilian National Health System and we were not able to access data from privately funded hospitalizations. However, recent estimates suggest that only 7.5% of older adults in Brazil have any form of private health insurance [34]. Therefore, our data is probably consistent with the health care provided to the majority of the older people in the city of Rio de Janeiro. Additionally, our limited database did not allow for a comparison between statistical models using previous hospitalizations and their main diagnoses as proxies for the burden of comorbidity of patients with models using more complete data about secondary diagnoses of patients. Finally, we were not able to analyze long term mortality outcomes beyond the hospitalization period because this would require having access to the Mortality Information System database, which was beyond the scope of this manuscript.

In conclusion, our results suggest that, in settings where local hospital information systems have limited access to secondary diagnoses, the use of the number of previous hospitalizations or the main diagnoses associated with those hospitalizations as proxies for the profile of comorbidities of older adults with hip fractures may not be an effective way to adjust for case mix when comparing in-hospital mortality rates among hospitals.

**References**

1. Donabedian A (1980) The definition of quality and approaches to its assessment. Health Administration Press, Michigan.
2. Travassos C, Noronha JC, Martins M (1999) Hospital mortality as an indicator of clinical performance: a review.Ciênc. Saúde Coletiva 4: 367-381.
3. Dubois RW, Rogers WH, Moxley JH 3rd, Draper D, Brook RH (1987) Hospital inpatient mortality. Is it a predictor of quality? N. Engl. J of Med 317:1674-80.
4. Blunberg MS (1988) Measuring surgical quality in Maryland: a model. Health Affairs 7: 64-78.
5. AHRQ Quality Indicators—Guide to Prevention Quality Indicators: Hospital Admission for Ambulatory Care Sensitive Conditions. Rockville, MD: Agency for Healthcare Research and Quality, 2001. AHRQ Pub. No. 02-R0203.
6. Hu F, Jiang C, Shen J, Tang P, Wang Y (2011) Preoperative predictors for mortality following hip fracture surgery: A systematic review and meta–analysis. Injury 45: 676-685.
7. Ireland AW, Kell PJ, Cumming RG (2015) Risk factors profiles for early and delayed mortality after hip fracture: analyses of linked Australian Department of Veterans’ Affairs databases. Injury 46: 1028-1035.
8. Endo A, Baer HJ, Nagao M, Weaver MJ (2018) Prediction Model of In-Hospital Mortality After Hip Fracture Surgery. J Orthop Trauma 32: 34-38.
9. Friesendorff M von, McGuigan FE, Wizert A, Rogmark C, Holmberg AH et al (2016) Hip fracture, mortality risk, and cause of death over two decades.Osteop. Intern 27:2945-2953.
10. Meyer HE, Tverdal A, Falch JA, Pedersen JI (2000) Factors associated with mortality after hip fracture. Osteop. Intern 11:228-232.
11. Aigner R, Buecking B, Hack J, Eschbach D, Oberkircher L, Ruchholtz S, Bliemel C (2017) Pre-fracture hospitalization is associated with worse functional outcome and higher mortality in geriatric hip fracture patients. Arch Osteoporos.32:2-6.
12. Green J, Wintfeld N, Sharkey P, Passman LJ (1990) The importance of severity of illness in assessing hospital mortality. J Am Med 263: 241-246.
13. Iucif NJR, Rocha JSY (2004) Study of inequalities in hospital mortality using the Charlson comorbidity index. Rev. Saúde Pública 38:780-786.
14. Martins M, Travassos C, Noronha JC (2001) Brazilian Hospital Database System as risk adjustment in performance indicators. Rev. Saúde Pública. 35:185-192.
15. Marufu TC, White SM, Griffiths R, Moonesinghe SR, Moppett IK (2016) Prediction of 30-day mortality after hip fracture surgery by the Nottingham Hip Fracture Score and the Surgical Outcome Risk Tool. Anaesthesia. 71: 515-521.
16. Zeeland ML, Genovesi IP, Mulder JW, Strating PR, Glas AS, Engel AF (2011) POSSUM predicts hospital mortality and long-term survival in patients with hip fracture. J Trauma.70(4): E67 - 72.
17. Pugely AJ, Martin CT, Gao Y, Klocke NF, Callaghan JJ, Marsh L (2014) A risk calculator for short-term morbidity and mortality after hip fracture surgery. J Orthop trauma. 28:63-69.
18. Jiang HX, Majumdar SR, Dick DA, Moreau M, Raso J et al (2005) Development and initial validation of a risk score for predicting in-hospital and 1-year mortality in patients with hip fractures. J Bone Min Resear 20:494 – 500.
19. Pinheiro RS, Coeli CM (2007) Sistema de informações hospitalares do SUS: aprimoramento da qualidade e inclusão de campos adicionais. Cad. Saúde Colet.15:167-168.
20. Camargo KR Jr., Coeli CM (2000). Reclink: aplicativo para o relacionamento de bases de dados, implementando o método probabilistic record linkage. Cad. Saúde Pública.16(2): 439-447.
21. Camargo KR Jr., Coeli CM (2015). Going open source: some lessons learned from the development of OpenReclink. Cad. Saúde Pública.31(2): 257-263.
22. Coutinho ES, Coeli CM (2008) Accuracy of the probabilistic record linkage methodology to ascertain deaths in survival studies. Cad Saúde Publica. 22:2249–52**.**
23. Charlson ME, Pompei P, Ales KL, Mackenzie CR (1987) A new method of classifying prognostic comorbidity in longitudinal studies: development and validation. J. of Chronic Dis 40. 373-83.
24. Souza RC, Pinheiro RS, Coeli CM, Camargo JKR (2008) The Charlson comorbidity index (CCI) for adjustment of hip fracture mortality in the elderly: analysis of the importance of recording secondary diagnoses. Cad. Saúde Pública. 24: 315-322.
25. Pinheiro RS, Vieira RA, Coeli CM, Vidal EIO, Camargo KRJr (2016) Mortalidade após fratura proximal de fêmur. Cad. Saúde Coletiva. 14(2):327-336.
26. Souza RC, Pinheiro RS, Coeli CM, Camargo JKR, Torres TZG (2007) Aplicação de medidas de ajuste de risco para a mortalidade após fratura proximal de fêmur. Rev. Saúde Pública 41: 625-631.
27. Garcia R, Leme DL, Leme LEG (2002) Evolution of brazilian elderly with hip fracture secondary to a fall. Clinics. 61:539-544.
28. Sakaki MH, Oliveira AR, Coelho FF, Leme LEG, Suzuki I et al (2004) Study of the proximal femoral fractures mortality in elderly patients. Acta Ortop. Bras 12: 242-249.
29. Vidal EIO, Moreira-Filho DC, Pinheiro RS, Souza RC, Almeida LM, Camargo Jr. JR, Villas Boas PJF, Fukushima FB, Coeli CM (2014) Hip fracture in a developing country: a picture in need of change. Journal of Aging Research & Clinical Practice. 3:182-188.
30. Rath S, Yadav L, Tewari A, Chantler T, Woodward M, Kotwal P, Jain A, Dey A, Garg B, Malhotra R, Goel A, Farooque K, Sharma V, Webster P, Norton R (2017) Management of older adults with hip fractures in India: a mixed methods study of current practice, barriers and facilitators, with recommendations to improve care pathways. Arch Osteoporos. 55:1-13.
31. Cree M, Soskolne CL, Belseck E, Hornig J, McElhaney JE et al (2000). Mortality and institucionalization following hip fracture. J Am Geriatr 48:283-288.
32. Gdalevich M, Cohen D, Yosef D, Tauber C (2004) Morbidity and mortality after hip fracture: the impact of operative delay. Arch Orthop Trauma. Surgery 124:334-340.
33. Folbert EC, Hegeman JH, Vermeer M, Regtuijt EM, Velde D van der et al (2017) Improved 1-year mortality in elderly patients with a hip fracture following integrated orthogeriatric treatment. Osteop. Intern 28: 269-277.
34. ANS Caderno de Informação da Saúde Suplementar: Beneficiários, Operadoras e Planos. Rio de Janeiro, RJ: Agência Nacional de Saúde Suplementar. ANS. 2017. Pub. No 02: 1-64.
