# Supplementary material for: Is the number of previous hospitalizations associated with increased in-hospital mortality after hip fracture in a developing country?
Source: PLoS One. 2020 Oct 9;15(10):e0240229. doi: 10.1371/journal.pone.0240229 (PMC7546455; doi:10.1371/journal.pone.0240229)
Supplement: Attachment — Submitted filename: Response to Reviewers_06102019.docx [file pone.0240229.s003.docx]

**PLOS ONE**

Rebuttal Letter

Manuscript Title: **Is the number of previous hospitalizations associated with increased in-hospital mortality after hip fracture in a developing country?**

Dr. Felipe Hada Sanders

Academic Editor

PLOS ONE

October 3, 2019

Dear Dr. Sanders,

We are very grateful for the opportunity you have given us to review our manuscript and for your careful assessment of our work. Please, find below our point-by-point responses to yours and the reviewer’s comments. We hope that you will find the new version of our manuscript represents an improvement over the first version and that you may consider it suitable for publication.

Sincerely,

Helen Regina Mota Machareth de Morais

**Journal Requirements**

1. Please ensure that your manuscript meets PLOS ONE's style requirements, including those for file naming. The PLOS ONE style templates can be found at http://www.journals.plos.org/plosone/s/file?id=wjVg/ PLOSOne_formatting_sample_main_body.pdf and http://www.journals.plos.org/plosone/s/file?id=ba62/ PLOSOne_formatting_sample_title_authors_affiliations.pdf

Authors' response:

We revised the manuscript to comply with PLOS ONE's style requirements including those for file naming.

2. In ethics statement in the manuscript and in the online submission form, please provide additional information about the patient records used in your retrospective study. Specifically, please ensure that you have discussed whether all data were fully anonymized before you accessed them and/or whether the IRB or ethics committee waived the requirement for informed consent. If patients provided informed written consent to have data from their medical records used in research, please include this information.

Authors' response:

In the revised version of our manuscript we have provide more detailed information regarding the patients’ records used in our study. The new section of our manuscript dedicated to the ethical approval of our investigation reads as follows:

“The Ethics Review Committees of the Public Health Institute and the Municipal Health Secretary approved this study under the processes 44114515.7.0000.5286 and 15903313.0.3001.5279, respectively. Following Brazilian regulation for ethics in research the ethics committees waived the requirement for informed consent because obtaining informed consent would have been impossible or impracticable since this was an observational retrospective study based on secondary data from patients who were no longer under follow-up at the evaluated hospital units. The probabilistic record linkage process was carried out using identified databases because that method [20-21] required the names of patients as an important source of information for the linkage between databases. Information was anonymized and de-identified prior to analysis. Only the research team had access to those databases, which were stored in a secure server at the Public Health Institute.”

**Additional Editor Comments**

beautifully written. please attach the ethics comittee approval.

Authors' response:

Thank you for your generous comments. We have uploaded the reports with the approval of our study by both Ethics Review Committees to the Editorial Manager Submission System.

Please note that we have added a data availability statement to the end of the manuscript explaining that the authors do not have the permission to deposit the datasets used in this study into public domain because even the de-identified dataset derived from the probabilistic record linkage process has the potential to be re-identified through the combination of several databases. If other researchers wish to have access to the de-identified dataset, they should contact the authors who will file an authorization request to the Ethics Research Committees that authorized this study. Once that permission is granted by both Ethics Research Committees the authors shall be able to share the study dataset with requesting researchers.

**Reviewer’s Comments**

P.9, line 110 of the attached file: “Please attach this approval”

Authors' response:

We have uploaded the reports with the approval of our study by both Ethics Review Committees to the Editorial Manager Submission System. Please note that we have also corrected the identification numbers of both processes at each Ethics Review Committees.
